# The M.A.G.I.C. framework for mHealth development: applying game design principles from ‘Magic: The Gathering’

**DOI:** 10.3389/fpsyg.2026.1675801

**Published:** 2026-04-10

**Authors:** James Broussard, Jana Fleet, Joseph McBride, Shanteria Brock, Joanne LeBrun, Monica Miller, Edelgard Wulfert

**Affiliations:** 1 Department of Psychology, Jackson State University, Jackson, MS, United States; 2 Department of Psychology, University at Albany, State University of New York, Albany, NY, United States

**Keywords:** app development, behavioral health, digital health technologies, digital therapeutics, game design, gamification, mHealth, user engagement

## Abstract

With the rapid advancement of digital health technologies, developers and behavioral health experts are increasingly exploring unconventional methods to modernize technology-based clinical interventions. The primary aim of this paper is to outline game design principles and lessons described by the developers of Magic: The Gathering (MTG), one of the world’s most popular and enduring fantasy role-playing card games, and explore how these tenets can be used to enhance mHealth (including wellness apps, wearable devices, and digital therapeutics). To accomplish this aim, we synthesized 20 lessons compiled by Mark Rosewater, the head designer of MTG, into a framework to aid development of digital health interventions, called the M.A.G.I.C. framework for mHealth. Key components of this framework include Mesmerization, Audience-centeredness, Goal-orientation, Individualization, and Community-drivenness. These elements, which helped to make MTG a worldwide success, can be used to create engaging, customizable, and user-friendly mHealth interventions. Thus, the M.A.G.I.C. framework for mHealth provides a template for mHealth development that encourages user engagement and maximizes the potential for enhanced therapeutic outcomes.

## 1 Introduction

Digital health technologies and mobile health (mHealth) have become a central feature of the behavioral health landscape, offering users support for anxiety, depression, stress management, and emotional regulation through scalable, provider-delivered and self-guided tools (Firth et al., 2017; Luxton et al., 2011). Despite the proliferation of mHealth technologies such as wellness apps, wearable devices, and digital therapeutics (American Psychological Association, 2025) and widespread public interest in accessible mental health resources, user engagement remains a chronic problem (Broussard and Teng, 2019). Many apps grounded in evidence-based practices, such as cognitive behavioral therapy (CBT) or mindfulness, are downloaded by users but suffer short lifespans due to inadequate customization, poor interface quality, and lack of sustained appeal; thus, they are rarely used beyond the first few sessions (Baumel et al., 2019; Krebs and Duncan, 2015; Krisher et al., 2024; Vaghefi and Tulu, 2019; Vo et al., 2019; Wu et al., 2021). Furthermore, evidence of the effectiveness of mHealth apps in terms of behavioral outcomes is limited, as efficacy studies are often based on small and possibly biased samples (Broussard and Teng, 2019; Linardon et al., 2024). This creates a paradox: we have tools that are potentially effective, but they are rarely used long enough or consistently enough to make a real difference.

Part of the problem is that many mHealth apps are designed first from a clinical or educational perspective. For example, poorly designed mHealth apps may present users with 20 slides of psychoeducational material followed by a quiz. While this might satisfy some clinical guidelines, it does little to encourage return visits or to foster a sense of progress. For this reason, some mHealth apps have deliberately incorporated game design elements such as points, badges, and leaderboards to increase motivation and engagement (Deterding et al., 2011). Examples include habit-forming apps like Habitica (HabitRPG Inc, 2025) which turn daily tasks into quests, awarding experience points for completing goals. Other apps for mindfulness and meditation, such as Headspace (2024), use streak counters, progress bars, and milestone animations to reinforce regular use. These apps demonstrate the appeal of gamification but also illustrate its limitations because they mainly use surface-level components without changing the structure or process of the experience. Tracking mood or completing therapeutic exercises may be clinically important, but simply awarding a badge for doing so does not change the experience itself. Users recognize the badge as meaningless, and their engagement quickly fades.

The gap between clinical potential and lived experience raises a fundamental question: what makes a digital therapeutic feel worth returning to? Efforts to “gamify” mental health tools by adding badges, points, or leaderboards to incentivize use fall short, resulting in what critics call the “chocolate-covered broccoli” problem (Habgood and Ainsworth, 2011). For example, a dull mood-tracking task that awards users a badge after 10 entries is still dull. The badge does not make the activity more engaging or therapeutically valuable. The broccoli (i.e., the task) is still dry and hard to chew, and the chocolate (i.e., points, badges) quickly wears thin. Users see through the artificial rewards and disengage from the core task, which remains uninteresting or overwhelming.

## 2 Therapeutic parallels: Where game design meets clinical practice

Games are among the most effective systems humans ever created for supporting sustained learning and growth (Irabor et al., 2025). Game mechanics are not just decorative; they are engagement engines. They create structured challenges, feedback, and rewards that guide player behavior over time. Digital health technologies can and should borrow from this logic, as the same mechanics can be used to structure therapeutic experiences. Rather than grafting game-like elements onto existing tools, we argue that mHealth can be meaningfully transformed by drawing on the deeper principles of successful game design.

Psychological theories support the parallel between game mechanics and therapeutic processes. According to Self-Determination Theory (Ryan and Deci, 2000), people are most motivated when their needs for competence, autonomy, and relatedness are met. Well-designed games excel at fulfilling these needs, offering challenges that feel achievable (competence), freedom to explore or customize (autonomy), and opportunities for social connection (relatedness). Therapeutic interventions, especially those based on cognitive behavioral therapy, are also most effective when they provide structured practice, promote personal insight, and allow for self-directed reflection (Hofmann et al., 2012). This convergence between psychological theory and game design reveals an untapped opportunity: therapeutic interventions can draw on the same principles that make games engaging to support lasting use.

To date, research on the use of game mechanics within a mental health context has been minimal (Deterding et al., 2011; Schmidt-Kraepelin et al., 2020). This gap often stems from the perception that games and therapy occupy different domains, one dedicated to entertainment, the other to healing. However, the growing field of serious games, defined as games that do not have entertainment, enjoyment, or fun as their primary purpose (Dereje et al., 2025), effectively bridges this divide. By demonstrating that both games and therapeutic interventions are architected to shape experience through exploration, reflection, and behavior change, serious games highlight the immense potential of interdisciplinary borrowing. Consequently, these design principles should serve as a rich source of inspiration for digital health technologies seeking to foster sustained engagement and meaningful psychological change.

## 3 “Magic: The Gathering” as a model for engagement

A powerful example of effective game design comes from the trading card game Magic: The Gathering (MTG; Wizards of the Coast, 2016a). MTG is a complex strategy card game that has sustained a global player base for over 30 years, making it one of the most successful card games ever created (Wizards of the Coast, 2016a, 2016b). In MTG, players represent powerful dueling wizards called Planeswalkers and engage in strategic gameplay using “mana” – a resource represented by different cards that feature spells, magical creatures, and special abilities – to defeat opponents (Elsam, 2019). The objective in MTG is to deplete the opponent’s power by reducing their life total to zero through a combination of strategic card play, which includes diverse attacks and other effects.

Since MTG’s debut in 1993, it has retained millions of players, sustained a vibrant tournament scene, and inspired countless spin-offs and design imitators. Furthermore, its popularity has accelerated in recent years with the release of “Magic: The Gathering Arena,” a digital version accessible on smartphones, tablets, and computers (Wizards of the Coast, 2019). What makes MTG remarkable is not just its fantasy theme or collectible format; it is the underlying system of rules, feedback, and progression that makes it inherently engaging. MTG exemplifies what makes a system deeply appealing: it keeps players engaged intellectually and emotionally through structured progression, provides meaningful choices with clear feedback, enables skills-based learning, allows for creativity and personal expression, and builds mastery over time through both individual and social play.

Many core features of MTG closely align with therapeutic mechanisms that are central to behavioral change. Both approaches aim to scaffold learning, reinforce feedback, and help individuals make better choices over time. By focusing on the parallels between game mechanics and therapeutic processes and formalizing them into a cohesive design framework, developers and behavioral health experts can collaborate to build mHealth technologies that are not only clinically effective but also offer users a journey that provides richness and depth of experience.

## 4 The M.A.G.I.C. framework for mHealth: An enhanced framework for mHealth design

At the 2016 Game Developers Conference, Mark Rosewater, the internationally famed head designer of MTG, gave a talk titled “‘Magic: The Gathering’: 20 Years, 20 Lessons Learned”, sharing 20 key lessons from his extensive experience in game design acquired throughout MTG’s history (Rosewater, 2016). For example, lessons include insights into the importance of aligning game mechanics with players’ natural tendencies. Rosewater stated that players must experience emotional connectivity with the game to enjoy both its intellectual and emotional stimulation. He also declared that games should be customizable, allowing each player to tailor the game to their own unique play style and preferences. Furthermore, he urged game developers to make the most effective strategies fun to engage in, thereby reinforcing optimal play.

We submit that applying Rosewater’s game design strategies to mHealth development for mental and behavioral health issues could inspire innovative approaches that engage users and encourage them to return. Thus, we sought to identify relevant parallels between MTG and mHealth development by identifying key features of Rosewater’s 20 lessons that contributed to MTG’s success. To aid in this synthesis, we used AI-supportive inductive thematic analysis (Perkins and Roe, 2024) to systematically identify key themes contained within two source documents: (1) a transcript of Rosewater’s talk “‘Magic: The Gathering’: 20 Years and 20 Lessons Learned” (2016), and (2) an integrative summary by the authors that related each of Rosewater’s lessons to therapeutic interventions and mHealth applications that use such features (source documents can be located in the Supplementary materials). Though this should not be construed as a qualitative study, we chose to use AI-supported inductive thematic analysis to ensure that all central themes between the two works were adequately captured.

Using the approach described above, five central themes of game design that are applicable to mHealth development were identified. These themes, which describe the core design features of the best-structured games, not just MTG, consist of (1) mesmerization, (2) audience-centeredness, (3) goal-orientation, (4) individualization, and (5) community-drivenness. These themes are not just compatible with the development of digital health technologies but can serve as a blueprint for design features that lead to truly transformative mHealth interventions. To help translate these parallels into action, we then formed these themes into the M.A.G.I.C. Framework for mHealth, which serves as an organizational structure for Rosewater’s 20 lessons. Each of the five pillars acts as a thematic bucket that synthesizes specific design principles from the original 20 lessons into actionable mHealth development strategies (see Table 1). In the following sections we will describe each of the key components of this framework to aid behavioral health experts and app developers in maximizing such features.

**Table 1:** The M.A.G.I.C. framework for mHealth: promoting playful and purposeful therapeutic design for mHealth.

Element	Key features
Mesmerization – The mHealth app or intervention should both evoke and elicit deep emotion and immersion through aesthetics and by reflecting the user’s established identity, values, and culture.	Reflect the user’s identity, values, and culture^3,8^ Evoke the intended emotion^6^ Enhance the aesthetics^2^
Audience-centeredness – The mHealth app or intervention should prioritize user experience, with clear, direct communication rather than showcasing advanced features.	Prioritize the user’s experience^12,15,19^ Make your message clear^14^ Embrace boldness^11,16^ Design with human nature in mind^1^ Remember that constraints spark creativity^18^
Goal-orientation – The mHealth app or intervention should integrate game elements and principles to enhance user motivation and skill acquisition.	Promote mastery through structured skill progression Incorporate dynamic feedback and adaptive challenge^16^ Balance intellectual and emotional engagement^5^ Align fun with the correct therapeutic strategy^13^ Leverage resonance for enhanced gamification^3^
Individualization – The mHealth app or intervention should be detailed and customizable, providing an experience that allows users to express and, ultimately, shape their identity by co-creating their therapeutic journey.	Offer opportunities for personalization, customization, and ownership^7,8,9^ Use resonance and piggybacking to catalyze action^3,4^
Community-drivenness – The mHealth app or intervention should foster a sense of social connectedness and belonging, facilitate interaction, and allow peers, clinicians, and support systems to innovate and share their experiences.	Foster a sense of community Nurture engagement through player-driven innovation^9,10^

Numbered lessons from Rosewater (2016) are included as superscripts. For Rosewater’s individually numbered lessons, see the Supplementary materials.

### 4.1 Mesmerization

Mesmerization—the intentional evocation and elicitation of emotion—is a core principle of effective app design. By engaging users’ identity, values, and cultural references, apps can create a dynamic and emotionally immersive experience. This requires a holistic design approach in which narrative, visual style, and interface elements work together to elicit a coherent emotional state (e.g., calm, motivation, excitement). When emotional resonance is consistent, apps feel more impactful, promote sustained engagement, and more effectively support behavioral health goals. The lessons related to mesmerization are outlined below.

#### 4.1.1 Reflect the user’s identity, values, and culture

Rosewater (2016) states that resonance is also about evoking emotion, explaining that designers can tap into players’ pre-existing emotional connections to concepts from pop culture, such as vampires and werewolves, to build on the emotional equity users already carry. MTG designers use experience-based resonance by carefully crafting each card’s graphics and design to foster a sense of fantasy and emotional connection for players. “As the player explores their choices, they are searching for things to bond with” (Rosewater, 2016). This idea is central in Acceptance and Commitment Therapy (ACT) (Hayes et al., 1999), where values clarification is not an afterthought but a foundation. Clients are guided to explore what truly matters to them, whether creativity, connection, compassion, or personal growth. Even difficult or painful steps become bearable when they serve a larger purpose aligned with the client’s identity. Digital therapeutics can harness the same power. The bond enhances their drive to continue using the therapeutic approach and makes the experience more immersive. When users see their identities, values, or aspirations reflected in a digital therapeutic, they are more likely to engage deeply. For example, users might choose avatars that represent personal strengths or reflect their aspirations (e.g., a wise mentor, a resilient warrior), or they might embark on narrative-based “quests” aligned with emotional goals such as self-compassion or courage. Another lesson commented upon by the designers of MTG is that not every detail will matter to every user, but the most minor detail may be significant to some. Thus, images, sounds, stories, and passages that reflect users’ emotions help them explore connections and form meaningful bonds with the mHealth app.

#### 4.1.2 Evoke the intended emotion

Rosewater (2016) stresses the need to understand what emotion a game is trying to evoke and ensure every element contributes to that feeling, whether it’s the “terror” of a horror-themed set or the fun of a humorous one. He states, “To be successful with your game, you need to know what your audience is trying to experience… everything in the game has to contribute to the emotional output you’re trying to create” (Rosewater, 2016). This principle emphasizes the importance of mHealth designers and behavioral health experts creating features that elicit emotional responses, guiding users toward their desired behavioral health outcomes. If an mHealth app aims to elicit positive emotions, its features should be designed to maximize these outcomes. Feelings of surprise, humor, and warmth can be fostered through text-based narratives, sound prompts, and visual design. Features should be consistently evaluated and refined to ensure they contribute to the intended emotional experience. A good example of this principle is the relaxation app Calm, which uses “soothing colors, balanced design elements, and gentle animations” to create a calming environment (Calm, 2024). By consistently eliciting feelings of peace and relaxation, Calm helps users manage stress and anxiety and moves them closer to achieving their mental health goals.

#### 4.1.3 Enhance the aesthetics

In MTG, the developers underscore the importance of aesthetics in game design and emphasize how ornamentation, symmetry, color, and overall design contribute to the player’s immersion in the experience. Rosewater says, “If you fail to provide the aesthetics you want, it makes the players feel ill at ease, it distracts them from the focus of your game, and it makes them pay attention to what the game isn’t instead of what the game is” (2016). Similarly, in behavioral health, aesthetics play a crucial role through the concept of phenomenology, the conscious process by which individuals perceive and interpret their lived experiences (Lazard et al., 2021; Koch, 2017; Picton et al., 2017). A well-designed interface with harmonious colors and clean lines, or one that allows users to select colors, fonts, and layout styles that resonate with their personal experience and emotional state, can enhance their comfort and their willingness to use the mHealth app regularly.

### 4.2 Audience-centeredness

Audience-centric design is crucial for creating effective mHealth apps. This understanding informs key design principles, such as ensuring messages are clear, embracing boldness to evoke a strong user response, using creative constraints to build superior features, and by designing interfaces that align with intuitive human behaviors. By tailoring the product to specific user profiles, this approach creates a personalized and engaging experience that effectively supports behavior change, rather than simply showcasing technology. The following sections discuss some of the key lessons relating to audience-centric design for mHealth.

#### 4.2.1 Prioritize your user’s experience

Development teams must design mHealth components with the target audience in mind, ensuring inclusion of elements that enhance the user experience and support behavior change. To achieve this, as illustrated by Rosewater, development teams can employ psychographics, a technique used in marketing to examine factors that drive consumer behavior (Barber et al., 2012). Thus, a digital therapeutic could utilize psychographic profiling to tailor content delivery to individual users or audiences. For example, development teams might create a profile for “Mindful Maeve,” a 32-year-old marketing coordinator who values holistic wellbeing, personal growth, and authenticity, seeking ways to manage stress and anxiety. She is conscientious, open to new experiences, and is primarily motivated by a desire for emotional regulation, consistency in healthy habits, and accessible tools to reduce stress and track her progress. Teams can contrast this with a profile for “Gamified Gary,” a 25-year-old software developer driven by challenge, competition, and a desire for tangible rewards and recognition. Whereas Maeve is easy-going and accepting of certain app limitations, Gary is highly data-oriented, enjoys optimizing his performance, and is seeking an mHealth app that offers daily challenges, leaderboards, virtual badges, and quantifiable progress tracking to maintain his mental health goals. Designing with these profiles and others in mind will allow teams to tailor their product to the anticipated audience for the app.

Additionally, designers should consider using other methods such as focus groups, website analytics, and social media activity to understand player profiles. Implementing iterative testing and feedback loops can help refine the therapeutic based on real user experiences (Huguet et al., 2023). Beta testing with a diverse group of users allows for collecting feedback on usability, functionality, and overall satisfaction (Cho et al., 2024). Continuous updates and improvements based on this feedback ensure that the therapeutic evolves to meet user needs effectively and focuses on delivering the best possible experience rather than just showcasing advanced features. By continuously analyzing user behavior and feedback, the digital therapeutic can provide a personalized experience that aligns with the specific needs and preferences of the intended audience.

#### 4.2.2 Make your message clear

Rosewater (2016) likens bluntness to a hammer in one’s creative toolbox, emphasizing its effectiveness in delivering a clear and directed message. In contrast, vague information can lead to various interpretations, potentially obscuring the intended message, and subtlety may result in critical information being overlooked. Therefore, when designing mHealth applications, developers and behavioral health experts should prioritize clarity to ensure users fully comprehend the information, objectives, or tasks presented. Instead of lengthy articles, the mHealth intervention might use bullet points, infographics, and short videos to convey essential information about mental health conditions and treatments efficiently. This approach ensures that users quickly grasp important concepts without feeling overwhelmed, thus becoming more able to improve their knowledge and confidence in managing their health.

#### 4.2.3 Embrace boldness

Rosewater states, “When you bore the players, they resent you. Sometimes they stop playing. So, as game designers, I think we have it reversed. Challenging the players isn’t the bigger threat. The greatest risk is not taking risks [so] stop worrying about evoking a negative response and start worrying about evoking a strong response” (Rosewater, 2016). mHealth has tremendous potential for unique and transformative behavioral health interventions not possible with traditional clinical methods (Jeon et al., 2024). Also, mHealth can present established interventions in more dynamic and appealing ways. Traditionally driven by creating gold-standard treatments with proven clinical effectiveness, the behavioral health field often progresses slowly. In contrast, the rapid release of new mHealth apps offers users a wide range of options, though it can also lead to difficulties in selecting the most beneficial apps (Wasil et al., 2021). While novel approaches require rigorous testing to determine acceptability, feasibility, and efficacy, instead of viewing this as a barrier, the creative potential of diverse applications should be harnessed to provide users with novel treatment options that often may not be available with traditional behavioral health treatment.

#### 4.2.4 Design with human nature in mind

Rosewater (2016) explains that fighting against human nature is a losing battle. He recounts how game designers initially prevented a creature card from attacking immediately after being played, a rule that players instinctively ignored. The eventual solution was to change the rules to align with the players’ natural tendencies. Thus, he advises that games should be designed to align with the natural tendencies of their players. If smartphone users are accustomed to swiping to navigate, an mHealth app should incorporate these familiar gestures rather than forcing users to learn new, non-standard controls (Lazard et al., 2021). Designers might even incorporate instinctual behaviors like the “pushing away” of an aversive stimulus using swipe features, thus making the app more intuitive. By embracing and integrating these innate user behaviors, mHealth apps can create a more intuitive, engaging, and ultimately effective experience for promoting health and wellbeing.

#### 4.2.5 Remember that constraints spark creativity

Rosewater argues that restrictions breed creativity because constraints force designers to solve problems in novel ways. For example, he contrasts the difficulty of responding to an open-ended prompt as compared to a themed one, where the constraints push him down new creative paths. Similarly, mHealth developers might at times create superior features by narrowing their focus. When development teams attempt to address behavioral health issues by relying on accepted practice or customs to guide their decisions, rather than attempting to think outside of the box, they may inadvertently constrain themselves and their product. For instance, a development team might concentrate on perfecting a single feature, like a meditation guide with biofeedback, rather than rehashing an entire treatment protocol in app form. Alternatively, a team might restrict its focus to reducing a single symptom rather than trying to address an entire cluster of symptoms, allowing for more detailed and tailored tools.

### 4.3 Goal-orientation

MTG offers a vibrant example to guide gamification approaches for mHealth and other digital health interventions, providing users with specific goals that enhance competence (i.e., mastery) or demonstrate it relative to others (i.e., performance); both of which are central to goal orientation theory (Kaplan and Maehr, 2007). Indeed, MTGs enduring success stems from its fantasy aesthetic and the way it blends structure with freedom, offering players clear rules and systems while allowing for vast creativity and evolution. The game meets players where they are, challenges them at the edge of their abilities, and rewards growth, not unlike an effective therapy process. Both rely on feedback loops, encourage skill-building, support autonomy, and foster a sense of meaningful progress over time. The following sections discuss how mHealth interventions can benefit from each of the core elements of goal-orientation and gamification embodied by MTG.

#### 4.3.1 Promote mastery through structured skill progression

A central strength of MTG is how it supports mastery over time. Players begin with a basic understanding of the rules and card types. Through repeated play, they gradually learn to build more effective decks, anticipate opponents’ strategies, and refine their own decision-making through feedback and experience. As challenges increase in parallel with skill, players experience a growing sense of competence and control.

This progression closely parallels psychotherapy, where change is achieved through skill development rather than passive insight. In Cognitive Behavioral Therapy (CBT), for example, clients initially learn foundational skills such as identifying unhelpful thoughts or monitoring mood. With practice, these skills are combined and applied more flexibly—challenging cognitive distortions, engaging in behavioral activation, or preparing for emotional triggers (Beck, 2020). As in MTG, simple mechanics gradually give way to greater strategic depth.

An MTG-inspired mHealth app could encourage mastery by making progress visible and earned. Users might start with a limited set of tools (e.g., guided breathing exercises or thought-labeling prompts), with more advanced features introduced as engagement and understanding increase. Skill development could be organized through a therapeutic “growth map” or “skill tree,” allowing users to choose focus areas such as anxiety regulation, sleep improvement, or relationship building, while ensuring that each new layer builds on previously practiced skills. Framing exercises as brief challenges or narrative tasks can further promote active application. By positioning users as active participants in their own growth, this approach fosters a felt sense of competence and supports more durable behavior change (Peters et al., 2018).

#### 4.3.2 Incorporate dynamic feedback and adaptive challenge

MTG’s engagement is driven by dynamic feedback. Every decision—whether playing a card, blocking an attack, or selecting a deck archetype—produces immediate and observable outcomes. Strategies either succeed or fail in ways that can often be traced back to specific choices. This feedback is not merely evaluative; it is informative, supporting learning through a continuous cycle of action, outcome, and adjustment.

MTG also maintains engagement by carefully regulating difficulty. Players begin with simpler rules and cards, and gradually encounter more complex systems as their skills develop. Challenge is calibrated to stretch competence without overwhelming the player, keeping engagement within the “flow experience” (Kiili et al., 2012). A similar principle guides psychotherapy, where clinicians aim to keep clients within a zone of proximal development—challenged enough to grow, but not so challenged that they disengage (Wang and Xiang, 2022). This approach is central to cognitive behavioral therapy, which emphasizes real-world experimentation as a key path to insight, exposure therapy, where feared stimuli are introduced gradually, and to Dialectical Behavior Therapy (DBT), which emphasizes the stepwise development of distress tolerance and emotion regulation skills (Bennett-Levy et al., 2004; Foa and McLean, 2016; Linehan, 2025).

Effective mHealth apps should scaffold challenges in the same way. Early tasks might involve low-intensity activities such as guided journaling or grounding exercises, with progression toward more emotionally demanding interventions such as exposure tasks or behavioral experiments. Apps can prompt users to choose coping responses in real-world situations and then guide structured reflection (e.g., distress before and after, perceived effectiveness). Visual feedback, such as mood trends over time, can help users link actions to outcomes, while reflective prompts strengthen metacognition and psychological flexibility. As users gain confidence, increasingly complex scenarios can be introduced, mirroring how MTG players adapt to stronger opponents or new card sets. These feedback-driven experiences support self-efficacy through mastery experiences (Bandura et al., 1975) and facilitate belief change grounded in lived experience (Broussard and Teng, 2019).

#### 4.3.3 Balance intellectual and emotional engagement

While “informative” content is important for mHealth apps, developers and behavioral health experts should carefully attend to elements that resonate with users’ emotions to evoke both mastery and satisfaction. This is because mHealth apps lack the dynamic interaction found in provider-delivered interventions. To address this, mHealth designers and behavioral health experts must consider features that stimulate both intellectual and emotional responses. Different gamification features in MTG, including card mechanics, strategic elements, and interactions between these features, allow for the combination of intellectual stimulation with fun. Rosewater states, “your game can speak to your audience on an intellectual level, or it can speak to them on an emotional level. Now, both are valuable, but when you speak to the players on an emotional level, you are more likely to create player satisfaction” (2016). This balance ensures that the digital therapeutic is educational and enjoyable.

#### 4.3.4 Align fun with the correct therapeutic strategy

Rosewater describes creating a humorous game set where the optimal strategy became decidedly unfun: to win, players had to avoid talking or laughing to prevent their opponents from gaining an advantage. The designers recognized the drawbacks of this mechanic during play testing, and the lesson learned was to “make the fun part also the correct strategy to win” (Rosewater, 2016). There is an implicit agreement between game designers and players that if the player does what the game tells them to do, it will be an enjoyable experience. In other words, “fun cannot be tangential” (Rosewater, 2016). Even challenging psychological interventions offer opportunities for humor, warmth, and fun moments. Though psychotherapeutic and mHealth interventions are able to evoke behavior change through negative reinforcement (i.e., reductions in symptoms), the crucial drawback of mHealth is that digital interventions lack a provider to offer encouragement and praise (i.e., positive reinforcement) throughout the more challenging phases of treatment. Thus, the positively reinforcing aspects of mHealth interventions must be fittingly assigned, easily accessible, and integrated into the core therapeutic processes. Gamification features should provide opportunities for users to derive some level of enjoyment and satisfaction throughout their journey, and even more so when they are on the right track. This gamification principle ensures that engaging with the app’s core therapeutic purpose is also a rewarding and satisfying experience.

#### 4.3.5 Leverage resonance for enhanced gamification

Resonance refers to the ability of a game component to elicit emotional connectivity within an individual, which, according to Schell (2015), can be achieved in two ways: experience-based or truth-based resonance. Experience-based resonance allows users to engage with their fantasies and desires through the game, while truth-based resonance involves aligning with the user’s personal beliefs and values. Rosewater emphasizes the importance of resonance to enhance learning and increase engagement with games, such as using familiar fantasy tropes, for example, zombies. Applying these principles to mHealth design involves harnessing the emotional resonance of the target audience to create more engaging and practical applications (Vichta et al., 2018). This is exemplified by Zombies, Run! (Six to Start, 2012), an app that gamifies exercise by blending storytelling with fitness goals. It is intuitive for users to want to escape from a horror character, and this familiar trope fosters learning and engagement with the target behavior. Users also follow a narrative, complete missions, and overcome obstacles as they run or walk, making the physical activity even more engaging. This application of gamification creates emotional resonance by transforming fitness into an immersive experience, thereby boosting user engagement and motivation.

### 4.4 Individualization

To foster deep engagement, app development should prioritize individualization by giving users a profound sense of autonomy, personalization, and ownership over their experience. This is achieved by allowing users to co-create their journey based on personal values, using resonant themes and familiar analogies to develop connection. By allowing the app to become an authentic extension of the user’s identity, this approach supports intrinsic motivation and facilitates more sustainable and meaningful psychological growth. In the following sections, lessons from Rosewater’s framework that align with the pillar of individualization are reviewed.

#### 4.4.1 Offer opportunities for personalization, customization, and ownership

One of the most engaging aspects of MTG is the freedom it offers players to express their identity through gameplay. Rosewater states, “The details are where the players fall in love with your game. I cannot stress this enough… people are individuals. They want to sort of come to the game and find something that they can claim to be their own, and that’s going to happen in the details” (Rosewater, 2016). MTG players do not just follow a single path to success; they choose their own path. They might build a fast-paced, aggressive red deck, a slow and strategic blue one, or something entirely unconventional based on personal aesthetics or thematic interest. Some gravitate toward lore-rich card sets or quirky combos that reflect their personality. This freedom to craft one’s approach fosters a sense of ownership, creativity, and emotional investment in the experience. The game does not tell players how to win; it gives them tools and lets them decide.

Rosewater explains that familiarity breeds preference, and preference enhances perceived quality: “The more your players feel the game is about them, the better their brain will think of it… so when their deck wins, they win because the deck [is] an extension of themselves” (2016). This freedom of expression mirrors one of the most potent drivers of motivation in psychotherapy: autonomy. According to Self-Determination Theory (Ryan and Deci, 2000), autonomy is one of three core psychological needs, alongside competence and relatedness, that must be satisfied for deep, sustained engagement. When people feel they are acting out of their own volition rather than following orders, they are more likely to persevere, take risks, and find satisfaction in the process. Similarly, Motivational Interviewing (Miller and Rollnick, 2012) emphasizes evoking a client’s own reasons for change, rather than imposing goals externally. Too often, apps assign goals, track behaviors, or offer pre-packaged interventions with little regard for the user’s individual narrative. Change is most effective when it feels personally meaningful. Lumosity (2024), an mHealth app designed to enhance memory, mental agility, and problem-solving skills, demonstrates this concept well. The app uses personalized activities that match the user’s abilities based on age and an initial fit test. By choosing preferred areas for improvement, users feel the app is tailored to their unique needs, fostering a more profound commitment. While structure is important, engagement deepens when users are invited to co-create their journey. Imagine an mHealth intervention that begins not with a symptom checklist, but with a values inventory: users select or articulate what matters most to them—such as being a good parent, expressing creativity, contributing to their community, or living with integrity. From there, the intervention might offer exercises, challenges, or reflections tailored to those values. For example, someone who values connection might be encouraged to reach out to a friend, reflect on relational patterns, or log moments of meaningful interaction. Someone driven by creativity might be nudged to use journaling, visual expression, or storytelling as a means of processing emotion.

Just as MTG offers different “paths to victory,” apps can provide multiple routes toward therapeutic growth. Rather than a linear path, users could choose between structured skills training, open-ended reflective prompts, or goal-oriented behavioral experiments, all framed within what feels meaningful to them. Even small choices, such as customizing the app interface, choosing a metaphorical “character” to accompany the journey, or selecting the tone of reminders, can subtly reinforce autonomy and personal relevance. From a psychological standpoint, this supports intrinsic motivation and fosters self-congruence, the alignment between action and identity. When therapeutic work feels like it belongs to the client rather than being imposed upon them, engagement becomes more authentic and sustainable. It also reduces resistance, a common barrier in both clinical and digital interventions. Ultimately, designing for autonomy is not simply about letting users “choose what they like;” it is about helping them clarify who they are and making that self-visible in the therapeutic process. Apps that recognize this can move beyond compliance-based models and become powerful tools for identity formation, values-driven action, and long-term psychological growth.

#### 4.4.2 Use resonance and piggybacking to catalyze action

In game design, resonance draws players in because the game is enjoyable and feels relevant to who they are. MTG achieves this through evocative artwork, thematic storytelling, and archetypal characters like dragons, healers, and tricksters, figures that tap into universal themes and personal fantasies. These anchors help players feel seen, immersed, and invested in the game world which can then serve as a compass for action. Kohut posited that individuals have a psychological need to connect with self-objects – people, historical figures, fictional characters, or ideas – that reflect their own experiences and behaviors (Kohut, 1971; Rabstejnek, 2020). Thus, resonance can be strengthened through relevant cultural or contextual elements to catalyze user action. An mHealth app designed for stress management might offer guided practices voiced in the user’s preferred language or frame coping strategies using familiar cultural metaphors. Similar to resonance, the concept of piggybacking leverages players’ pre-existing conceptual and procedural knowledge to more efficiently teach new information and skills (Paxton-Buursma and Walker, 2008). To explain complex psychological concepts using piggybacking, an app might use analogies or allegories of familiar everyday activities (e.g., comparing negative thought patterns to physical clutter that must be “tidied up”). These approaches aid users in quickly grasping new concepts by relating them to familiar tasks, making the app’s content easier to follow and act upon.

### 4.5 Community-drivenness

Community-driven features for mHealth aim to promote social experience and connection among users. The following sections examine how design choices can foster a sense of community, facilitate interaction, and allow users to share their experiences, which can be a powerful motivator and support mechanism.

#### 4.5.1 Foster a sense of community

While MTG is celebrated for its strategic complexity, its staying power is equally rooted in its social experience. Players come together in game stores, online communities, and tournaments not just to compete, but to connect, sharing strategies, trading cards, and building friendships. These social interactions foster a sense of belonging and play a crucial role in keeping players engaged over time. They also align with a core tenet of Self-Determination Theory (Ryan and Deci, 2000): people thrive when they feel connected to others. In gaming and therapy, meaningful change often happens through human connections, not in isolation. mHealth apps often overlook this dimension, offering largely solitary experiences. While legitimate concerns about privacy and risk may exist, the absence of social connection can limit motivation and undermine retention. Just as therapy benefits from a strong therapeutic alliance, users of mHealth apps can benefit from knowing they are not alone in their journey. Apps can create more engaging experiences by incorporating personalized community features where users connect with others who share similar challenges. Elements like discussion forums, group challenges, and peer support systems can build a sense of belonging and community as well as facilitate user motivation, medication compliance, and treatment adherence by fostering supportive networks and optimal social comparisons (Cahn et al., 2018). Shared “goal walls” or timelines could promote gentle accountability, while collaborative challenges or “community quests” (e.g., a group logging a collective 1,000 min of mindfulness) can foster purpose and connection. This socialization can facilitate motivation and treatment adherence by creating supportive networks for users, which provides emotional support and increases satisfaction with the mHealth app. Incorporating optional, well-designed, and ethically moderated social features can also enhance engagement and behavioral health outcomes. For example, anonymous community boards allow users to share goals, offer encouragement, or tell their stories, normalizing struggle and celebrating progress. Even asynchronous social exchanges, such as viewing user-submitted reflections or responding to daily prompts, can increase motivation and create a subtle sense of presence and support. As MTG play groups provide a space for learning, belonging, and shared excitement, peer networks within mHealth can help users feel seen and supported. In domains where social interaction with other users might violate privacy or anonymity (or would be otherwise impractical), chatbots and AI-avatars can be used to simulate social processes. Thus, designing for social connection does not mean compromising privacy; it means recognizing that growth is often relational. With thoughtful implementation, digital health technologies can become more than tools; they can become communities of healing and shared purpose.

#### 4.5.2 Nurture engagement through player-driven innovation

Rosewater (2016) discusses the creation of Commander, a popular game format not designed by the company but by a group of players (i.e., MTG tournament judges) who wanted a unique way to play together after their work was done. This player-driven format was so popular that the company eventually began creating products to support it. This illustrates how allowing space for socialization and community-driven innovation can lead to deep and lasting engagement. Rosewater reiterates, “If you want your players to bond as closely as you can, the game has to move beyond being yours and become theirs. You have to find a way to make sure that the player can do that, and the key is customization, because you want your player to be able to do something that nobody else could do that they did, that’s their creation” (2016). This is closely aligned with the concept of leaving room for players to explore a game to make it their own. By providing a wide range of customizable content allowing discovery and user-driven design, mHealth apps can cater to the desires and needs of entire cross-sections of users, empowering them and fostering a sense of ownership.

## 5 Discussion

The convergence of game design and digital health technologies is not just a creative opportunity but a clinical necessity. In a digital mental health landscape flooded with apps that often are underused or quickly abandoned, there is a growing urgency to rethink what these tools are and how they engage people. Apps cannot simply rely on providing psychoeducation or logging symptoms. They must motivate, teach, adapt, and resonate emotionally to be effective. In short, they must do what the best games have always done.

The M.A.G.I.C. Framework for mHealth is a practical framework for designing mHealth technologies that move beyond layering superficial points or prizes onto pre-existing content. The framework emphasizes deep alignment between effective therapeutic processes and compelling game mechanics as typified by MTG and its developers’ insights. First and foremost, a well-designed mHealth app should seek to mesmerize the user, not through the use of gimmicks, but through emotional and cognitive connection. A compelling mHealth app should use visuals, sound, feedback, and narrative, not as decoration, but as tools that sustain attention and foster immersion and emotional connection. When users are pulled into a story, challenged by a task, or moved by a character or metaphor, they are more likely to return. For example, an app rooted in Acceptance and Commitment Therapy might guide users to clarify personal values, like connection, growth, or creativity, and then shape in-app quests and rewards around those values. This way, tasks become more than exercises; they become reflections of identity. Second, the app must be audience-centric. To create effective mHealth apps, development teams should prioritize the user’s experience, employing psychographic profiling and iterative feedback to tailor content. Additionally, teams should seek to provide clarity in communications, embrace boldness, and allow for creative risks in design, as constraints can paradoxically spark innovation. Third, and critically important, is that users’ experiences within the context of mHealth are goal-driven. Like any compelling game, it must offer structure and support, especially early on. Users need to know what to do next, and why. Apps should scaffold complexity, introducing skills gradually and giving users a sense of direction without overwhelming them. Just as game players learn core mechanics before tackling advanced strategies, users should begin with manageable therapeutic tasks before progressing to more challenging interventions. Fourth, users must be given opportunities to individualize their experience. Users need to feel that what they do inside the app connects to who they are on the outside. Just as players in MTG build decks that reflect their unique interests and characteristics, users should be able to define their own goals and choose paths that align with what matters to them. Thus, while mesmerization focuses on the initial immersion by aligning with the user’s current self-concept, individualization facilitates long-term engagement by providing the tools for identity transformation. Finally, mHealth apps must be community-driven, either through mechanisms such as message boards and sharing with peers or, when not possible, by encouraging users to seek social supports on their own or by providing users with AI-driven tools that simulate social processes. This is especially important for mHealth apps that seek to serve as stand-alone or self-help interventions for behavioral health issues.

The M.A.G.I.C. framework is not a formula, but a flexible lens, a way to help developers and behavioral health experts build tools that speak to both the science of behavior change and the art of play. However, achieving this requires more than good intentions. It demands collaboration. Clinicians understand the complexities of healing and the nuances of ethical care. Game designers and mHealth developers understand what makes an experience motivating, intuitive, and fun. Neither discipline alone can fully meet this challenge, but together, they can. Therefore, we must build interdisciplinary teams that combine clinical expertise and game design skills to move the field forward. Programmers, game designers, behavioral health experts, and end-users must work side by side to ensure that therapeutic effectiveness and user engagement are treated as core priorities, not afterthoughts. We must include users in the design process, testing, and refining with real people, not just clinical proxies. Moreover, we must expand our metrics for success beyond symptom reduction alone, looking also at skill acquisition, sustained engagement, and users’ sense of personal growth and meaning. Mental health deserves tools that do not just work, but that are emotionally resonant and that matter. Furthermore, we must look beyond the clinical textbook and into the design playbook. Game developers have spent decades figuring out how to create experiences that people choose, return to, and grow from. It is time that we brought that wisdom into the service of healing. Imagine a digital therapeutic where users face dragons of self-doubt with skills they have developed across months of guided practice. Where someone faces down their fear not by clicking a checkbox, but by completing a narrative arc where they have built the courage, one small act at a time. Resilience is earned not in theory, but through challenges mastered in gameplay; this is where digital therapy becomes not just something users must remember to do, but something they look forward to because it feels alive, personal, and real.

## 6 Limitations and future directions

In this paper, we have offered a conceptual argument for integrating principles of effective game design, particularly those derived from MTG, into the development of mHealth apps. While this synthesis offers promising directions, it also has limitations that must be acknowledged, as well as opportunities for future empirical work and refinement.

First, the M.A.G.I.C. framework is theoretical. While grounded in established principles from both clinical psychology and game design, it has not yet been empirically validated in mHealth development or evaluated through controlled studies. Future research should operationalize each component of the framework, develop design prototypes, and assess their effectiveness through usability testing, user engagement metrics, and clinical outcomes research. This limitation presents a key opportunity: researchers can collaborate with development teams to create small-scale pilot studies followed by randomized controlled trials comparing apps designed with and without M.A.G.I.C. principles.

Second, our approach relies heavily on analogical reasoning, mapping the logic of game systems like MTG onto therapeutic mechanisms. While these parallels are conceptually compelling, they may not always translate directly into practice. The motivations, contexts, and constraints of therapy differ in important ways from gameplay. For example, while failure in a game can be motivating, repeated “failures” in a therapeutic context may increase self-criticism or disengagement without careful framing. Therefore, future research should explore boundary conditions: when and how game-derived principles support therapeutic goals and where they may need to be tempered or adapted to avoid unintended effects.

Third, mHealth apps and games are often designed for a tech-literate, younger demographic, potentially excluding older adults, individuals with disabilities, or those with limited digital access. Similarly, cultural values around therapy and play differ significantly across populations. Game metaphors that resonate with some users may alienate others. Future iterations of the M.A.G.I.C. framework should incorporate inclusive design principles, prioritize co-design with diverse users, and explore how playful therapeutic systems can reflect different cultural models of healing, growth, and motivation.

Fourth, using engagement techniques from game design in digital health technologies may also raise ethical concerns. There is a thin line between increasing engagement and exploiting attention. Developers must be transparent about using behavioral nudges, avoid manipulative mechanics (e.g., loot-box analogues), and preserve user autonomy. As the field evolves, ethical guidelines, perhaps modeled on the ethics code of the American Psychological Association (APA) or the Association for Computing Machinery (ACM), should be developed to govern the design of persuasive health technologies that draw on game design principles.

Finally, although we argue for interdisciplinary collaborations between game designers, behavioral health experts, and clinicians, such partnerships can be challenging to initiate and maintain. Differences in language, incentives, timelines, and design cultures often make cross-disciplinary work slow or frustrating. Institutions and funding agencies can help address this limitation by supporting shared platforms, joint training programs, and seed funding for collaborative digital health projects. Building a shared vocabulary between disciplines will be key to unlocking the full potential of this integration.

## 7 Conclusion

Despite the challenges described above, the opportunities for digital health technologies, including mHealth, are immense. By building on a robust conceptual foundation and rigorously exploring its application, the next generation of mHealth technologies can not only be more effective, but more humane, offering users experiences that are structured, adaptive, personally meaningful, and deeply engaging. It is at the intersection of game design and psychological science that the future of digital health technology may truly begin to take shape.

## Data Availability

The original contributions presented in the study are included in the article/Supplementary material, further inquiries can be directed to the corresponding author.
