# Supplementary material for: The M.A.G.I.C. framework for mHealth development: applying game design principles from ‘Magic: The Gathering’
Source: Front Psychol. 2026 Apr 10;17:1675801. doi: 10.3389/fpsyg.2026.1675801 (PMC13106199; doi:10.3389/fpsyg.2026.1675801)
Supplement: Supplementary file 1 [file Data_Sheet_1.pdf]

Supplementary Material

1 Summary of “Magic: the Gathering' 20 Years, 20 Lessons Learned” (Rosewater, 2016)

What follows is a summary of each of the 20 lessons offered by Mark Rosewater’s during his presentation at the 2016 Game Developers Conference. The video of the presentation along with a full transcript can be obtained on the GDC’s YouTube channel (link included in the following reference):

Rosewater, M. (2016, May 16). *Magic: The Gathering: 20 years, 20 lessons learned* [Video]. YouTube. https://www.youtube.com/watch?v=QHHg99hwQGY

Lesson #1: Fighting Against Human Nature is a Losing Battle

"Don't change your players to match your game; change your game to match your players" (Rosewater, 2016).

Rosewater explains that players come with a "complex operating system" of inherent behaviors and psychological quirks. In the case of the *Suspend* mechanic, players instinctively wanted to attack with creatures the moment they entered the battlefield, despite rules forbidding it. Rather than continuing to fight this instinct, the designers eventually changed the rules to allow the behavior, reinforcing that a game will suffer if it ignores the natural inclinations of its audience.

Lesson #2: Aesthetics Matter

"Aesthetics matter... it studies how humans perceive the world" (Rosewater, 2016).

This lesson focuses on human perception and the "science of beauty". Using the card *Griselbrand* as an example, Rosewater notes that players felt "ill at ease" because the card’s mana cost (8) did not match its other numerical attributes (7 power, 7 toughness, 7 life, 7 cards), failing to satisfy a human desire for patterns and symmetry. Designers must understand that if a game's components do not "feel right" aesthetically, it distracts players from the core experience.

Lesson #3: Resonance is Important

"Your audience comes to your game with a lot of stuff already there" (Rosewater, 2016).

Rosewater posits that designers do not have to start from scratch because audiences are "preloaded" with emotional responses to existing concepts. By using evocative themes like Gothic horror (vampires, werewolves, and zombies), *Magic* was able to tap into "emotional equity" that players already possessed from years of pop culture consumption. Building upon these pre-existing emotional connections creates a richer and more immediate game experience.

Lesson #4: Make Use of Piggybacking

"Piggybacking is a use of pre-existing knowledge to front-load game information to make learning easier" (Rosewater, 2016).

Resonance serves as a powerful teaching tool by allowing designers to bypass complex rule explanations. For example, the "Flying" mechanic is easy to teach because it matches a player's natural expectation of how flight works. Similarly, using "Plants" as stationary units in *Plants vs. Zombies* reinforces the rule that the units cannot move without needing a formal tutorial, as the concept is "planted" in the player's mind through common knowledge.

Lesson #5: Don't Confuse "Interesting" with "Fun"

"Don't confuse interesting with fun" (Rosewater, 2016).

There is a distinct difference between intellectual stimulation ("interesting") and emotional stimulation ("fun"). In the *Odyssey* set, designers created a mechanic that asked players to discard their hands to gain power, which was an "intellectual" subversion of strategy but proved to be emotionally unsatisfying because players simply wanted to play their cards. While both are valuable, designers should prioritize emotional response, as it is more likely to lead to long-term player satisfaction.

Lesson #6: Understand What Emotion Your Game is Trying to Evoke

"To be successful with your game, you need to know what your audience is trying to experience" (Rosewater, 2016).

Designers must identify the specific emotional output they want to achieve and ensure every component serves that goal. When designing the *Innistrad* set, the goal was to evoke "fear" and "dread," leading to mechanics like *Morbid* (triggered by death) and double-faced cards (representing scary transformations). If a mechanic or "scene" does not advance the intended emotional experience of the "movie," it must be cut.

Lesson #7: Allow the Player the Ability to Make the Game Personal

"The more the players feel the game is about them, the better their brain will think of it" (Rosewater, 2016).

A psychological quirk associates familiarity and personal connection with higher quality. By introducing "Full Art Lands" and offering a vast array of colors, characters, and factions, *Magic* allowed players to find elements that resonated with them personally. Providing choices—and the ability for players to express themselves through those choices—creates a "personal connection" that strengthens the player's bond with the game.

Lesson #8: The Details are Where Your Player Falls in Love with Your Game

"The details are where your player falls in love with your game" (Rosewater, 2016).

While a single detail might only appeal to a small percentage of people, for that group, it can mean everything. Rosewater cites the character *Fblthp*, a tiny homunculus in a piece of card art that became a massive community favorite despite the card itself being strategically weak. These "insignificant" details provide the specific hooks that individuals use to bond with the game on a deeper, more enduring level.

Lesson #9: Allow Your Players to Have a Sense of Ownership

"Allow your players to have a sense of ownership" (Rosewater, 2016).

To foster the strongest possible bond, a game must move from being "yours" (the designer's) to being "theirs" (the player's). This is achieved through customization, such as deck building, where players create something uniquely their own from thousands of options. When a player wins with a deck they customized, it feels like an "extension of themselves" rather than just a victory within the game's rules.

Lesson #10: Leave Room for Your Players to Explore

"Don't always show the players the things you want them to see, let your players find them" (Rosewater, 2016).

Borrowing from the art of "the pitch," Rosewater argues that people are more invested in things they initiate or discover themselves. By creating cards like *Summoner's Pact* and *Hive Mind* that interacted in unexpected ways, designers allowed players to "discover" a winning combo they hadn't explicitly intended. This "room to explore" ensures that discovery remains a key part of the player's personal investment in the game.

Lesson #11: If Everyone Likes Your Game, But No One Loves It, It Will Fail

"If everyone likes your game, but no one loves it, it will fail" (Rosewater, 2016).

A "blind date" that checks all the boxes but lacks passion will not lead to a second date; similarly, games need strong, positive responses to survive. It is better to have a card that half the players hate and half the players love than one that everyone simply finds "okay". Evoking a strong response—even a polarizing one—is necessary to make players truly fall in love with the experience.

Lesson #12: Don't Design to Prove You Can Do Something

"Don't design to prove you can do something" (Rosewater, 2016).

Designers must ensure their decisions serve the "optimal experience for the target audience" rather than their own ego or desire for self-satisfaction. The card *Tibalt* was designed as a two-mana Planeswalker simply to see if it could be done, but this restriction made the card too weak to be enjoyable for fans of that character type. Decisions should always prioritize the quality of the game over the designer's internal need to test themselves.

Lesson #13: Make the Fun Part Also the Correct Strategy to Win

"Make the fun part also the correct strategy to win" (Rosewater, 2016).

It is the designer's responsibility to place the "fun" where the player cannot help but find it while trying to win. In the set *Unhinged*, the "Gotcha" mechanic rewarded players for their opponents laughing or talking, which led to a "winning strategy" of being silent and bored. Because players will follow the game's instructions to reach a goal, the designer must ensure those instructions lead directly to an enjoyable experience.

Lesson #14: Don't be Afraid to be Blunt

"Sometimes to get your audience to understand, you have to be willing to embrace bluntness" (Rosewater, 2016).

While artists often prefer subtlety, players can easily miss the obvious. When players were too afraid to attack with the powerful *Ulamog's Crusher*, designers added a line of text literally forcing the creature to attack, which quickly "educated" players on how good it was. Bluntness is a "hammer" in the creative toolbox that is necessary when subtlety fails to communicate essential gameplay concepts.

Lesson #15: Design the Component for the Audience it is Intended For

"Design the component for the audience it is intended for" (Rosewater, 2016).

Trying to please everyone often results in pleasing no one. Rosewater uses "psychographics"—Timmy/Tammy (experience), Johnny/Jenny (expression), and Spike (proof)—to categorize different player needs. A card that tries to mix coin-flipping (Timmy) with balanced, skill-based outcomes (Spike) may end up being hated by both because it doesn't maximize the experience for either. Each component should be focused on the specific subgroup it was made for.

Lesson #16: Be More Afraid of Boring Your Players Than Challenging Them

"Be more afraid of boring your players than challenging them" (Rosewater, 2016).

Players will forgive a "grandiose" failure because they respect the attempt to do something awesome, but they will not forgive boredom. When "Split Cards" were first proposed, many within the company feared they didn't "look like Magic" and were too risky. However, the risk paid off because players loved the challenge to their expectations. The "greatest risk" in game design is actually the refusal to take risks.

Lesson #17: You Don't Have to Change Much to Change Everything

"You don't have to change much to change everything" (Rosewater, 2016).

Designers often make the mistake of adding too much complexity, much like adding too many "peas" to a dish. Instead of asking how much to add, designers should ask "how little do I need to add" to achieve a new experience. By shifting from encouraging "as many colors as possible" to "only two

colors," designers created a radically different player experience in *Ravnica* compared to *Invasion*, despite both having a multicolor theme.

Lesson #18: Restrictions Breed Creativity

"Restrictions breed creativity" (Rosewater, 2016).

The human brain is efficient and tends to solve problems using the same "neural pathways" it has used before. Restrictions force the brain to start from a new "vantage point," leading to innovative solutions and ideas that wouldn't emerge in an open-ended environment. By embracing themes or arbitrary constraints, designers can bypass their own habits and discover their most creative work.

Lesson #19: Your Audience is Good at Recognizing Problems and Bad at Solving Them

"Your audience is good at recognizing problems and bad at solving them" (Rosewater, 2016).

Like a patient at a doctor's office, players know better than anyone how they feel, but they lack the tools and expertise to provide the cure. Players are an excellent "barometer" for identifying when something in a game is "wrong" or "unfun". However, designers should take player-suggested solutions with a "grain of salt" because players do not understand the underlying limitations and technical tools of the designer.

Lesson #20: All the Lessons Connect

"All the lessons connect" (Rosewater, 2016).

While these principles were presented as twenty separate items for "easy digesting," they actually form a single "complex interconnected holistic view" of design. For instance, "exploring" (Lesson 10) makes "details" (Lesson 8) matter more, and a "sense of ownership" (Lesson 9) makes it easier to align with "human nature" (Lesson 1). Understanding how these lessons interact is the key to mastering the craft of game design.

2 “Magic: The Gathering’ and mHealth: Game Design Principles” (Broussard et al., n.d.)

In 2016, Mark Rosewater, head designer of Magic: The Gathering (MTG), a fantasy-based tabletop card game, gave a talk titled Magic: The Gathering: 20 Years and 20 Lessons to a crowd of game developers, artists, and business leaders during their annual Game Developers Conference (GDC, 2016). In this talk, Rosewater offered valuable insights from the previous two decades of development and expansions of MTG in the form of 20 key lessons. These lessons offer insights from MTG to inspire new ideas and broaden existing perspectives on game design principles. The following list includes a summary of each lesson, excerpts from Rosewater’s “Magic: The Gathering: 20 Years and 20 Lessons” as well as examples and potential applications of each lesson within the context of mHealth design.

2.1 “Fighting against human nature is a losing battle”

Mark Rosewater asserts that attempting to force users to alter their inherent behaviors for the sake of a game will lead to poor outcomes. Instead, he advises that games should be designed to align with the natural tendencies of their players (GDC, 2016). In game design, knowing one’s audience implies understanding user behavior and adapting the design to fit their natural inclinations, as this enhances user experience and engagement (Sandu, 2023). This concept applies equally to user-centered mHealth design (Torous et al., 2019).

To illustrate, users of mHealth apps often have ingrained habits of interacting with digital interfaces. If users commonly swipe left or right to navigate through content on their smartphones, mHealth apps should incorporate these familiar gestures for more straightforward user navigation. Forcing users to adapt to unfamiliar controls, such as nonstandard buttons or complicated multi-step processes, can lead to frustration and decreased usage (Lazard et al., 2021). Similarly, in the context of stress management apps, users may exhibit instinctual behaviors like “pushing away” an aversive stimulus on the screen. For example, if an app presents a stressful scenario, allowing users to swipe away or zoom out from the stressor would align with their natural impulse to remove or mitigate negative stimuli, making the app more intuitive and user-friendly.

2.2 “Aesthetics Matter”

In MTG, the developers underscore the importance of aesthetics in game design and emphasize how ornamentation, symmetry, color, and overall design contribute to the player’s experience. Similarly, in behavioral health, aesthetics plays a crucial role through the concept of phenomenology, a conscious process by which individuals perceive and interpret their lived experiences (Koch, 2017; Picton et al., 2017). Behavioral health designers can create more effective and engaging interventions by better understanding how users experience and engage with a mHealth app. Here are a few ways this concept can be applied in mHealth design:

The visual design of an app can have an emotional impact: Aesthetics in mHealth apps can significantly influence users' emotional responses and engagement (Lazard et al., 2021). For example, Calm (2024), a mHealth app designed to promote relaxation and mindfulness, incorporates soothing colors, balanced design elements, and gentle animations to create a calming environment for the user. These features also include visual cues that mirror breathing exercises or calming imagery that evolves based on user input (Calm, n.d.)

An app for individuals managing chronic pain could offer customizable visual themes or interfaces that users can adjust based on their preferences or current mood. Aesthetic considerations incorporated into mHealth design align with the principles of phenomenology and enhance the effectiveness of behavioral health interventions. A well-designed interface with harmonious colors and clean lines or a design that allows users to select colors, fonts, and layout styles that resonate with their personal experience and emotional state can enhance their comfort and willingness to use the app regularly. Designers can create more engaging, supportive, and personalized experiences that improve usability, user satisfaction, and treatment outcomes by focusing on how users perceive and interact with the app.

2.3 "Resonance is Important"

Resonance refers to the ability of a game component to evoke emotional connectivity within an individual, which, according to Schell (2015), can be achieved in two ways: experience-based and truth-based. Experience-based resonance allows users to engage with their fantasies and desires through the game, while truth-based resonance involves aligning with the user's personal beliefs and values. Designers of MTG utilize experience-based resonance by carefully crafting each card's graphics and design to foster a sense of fantasy and connection for the player (GDC, 2016).

In the initial stages of mHealth app design, comprehending the impact of resonance is crucial for enhancing user engagement and improving behavioral treatment outcomes. In psychotherapy, a similar concept is empathic resonance introduced by psychoanalyst Heinz Kohut in his Self-Psychology approach. Kohut (1971) posited that individuals have a psychological need to connect with self-objects – people, historical figures, fictional characters, or ideas – that reflect their own experiences and behaviors (Rabstejnek, 2015). Similarly, from a Rogerian Humanistic perspective, empathic resonance within the therapeutic alliance involves the therapist attuning to the client's verbal and nonverbal cues, such as body language, tone of voice, and emotional shifts to facilitate a deeper connection and trust.

Applying these principles to mHealth design involves harnessing the emotional resonance of its target audience to create more engaging and practical applications (Vichta et al., 2018). For example, to enhance experience-based resonance, a mHealth app could allow users to create personalized avatars and storylines that reflect their aspirations and interests. For instance, a fitness app might let users design avatars that resemble their ideal self-image and integrate story arcs that align with their fitness goals. This personalization helps users connect emotionally with the app and enhances their motivation to engage regularly. A prime example of this type of engagement is found in the app Zombies, Run! (Six to Start, 2012), where users can blend the art of storytelling with their personal fitness goals. As users run or walk, they follow a narrative that allows them to complete missions, overcome obstacles, and hear audios that unfold into larger storylines (Six to Start, 2012). Through this, the app creates emotional resonance by making fitness more engaging for the user while personalizing their experiences and boosting their motivation.

Integrating elements that reflect the user's culture and attitudes can also enhance emotional resonance. For example, a mHealth app designed for managing chronic conditions might offer localized content, such as culturally relevant health tips, language preferences, and dietary recommendations. By embedding cultural references and user-specific traits into the app, designers can create a more relatable and engaging experience for diverse user groups (Gonzalez et al., 2021; Woulfe et al., 2022).

Incorporating these principles into mHealth app development ensures that the application resonates with users on an emotional level. This will increase engagement, enhance the effectiveness of behavioral health interventions, and lead to better treatment outcomes and user satisfaction.

2.4 "Make Use of 'Piggybacking'"

Similar to resonance, app designers and clinicians must also understand the mechanics of piggybacking to engage users effectively. Piggybacking, borrowed from educational contexts, means acquiring new information and skills largely depends on pre-existing knowledge (Paxton-Buursma & Walker, 2008). The designers of MTG use piggybacking through concepts like miracle cards in Avacyn Restored by tapping into users' pre-existing knowledge to make the game more easily understood. These miracle or spell cards symbolize luck, allowing users to activate powers that might help them defeat opponents (Ziegler, 2024). Using familiar concepts like miracles, users can quickly grasp the cards' mechanics, apply what they have learned to new concepts, and strategically use them.

Applying piggybacking to mHealth apps, developers can incorporate familiar narratives or themes from popular culture, literature, or everyday life experiences to make a mHealth app more relatable. For example, an app designed to help users manage anxiety might use a storyline similar to a well-known hero's journey, where users overcome challenges and build resilience. To explain complex psychological concepts, the app could use analogies and comparisons to everyday activities. For example, an app teaching cognitive-behavioral techniques might compare negative thought patterns to physical clutter, encouraging users to "tidy up" their thoughts just as they would clean their living space. This analogy helps users quickly grasp new concepts by relating them to familiar tasks, making the app's content more engaging and easier to follow.

2.5 "Don't confuse interesting with fun"

Engagement with mHealth apps often lacks the dynamic interaction found in provider-delivered interventions. To address this, app designers and clinicians must consider features that spark both intellectual and emotional stimulation. Observing different card characteristics, functions, and interactions in MTG combines intellectual stimulation with fun. Similarly, while "informative" content is crucial for mHealth apps, developers and clinicians should also include elements that resonate with users, such as vignettes, guided activities, or virtual exercises (Broussard & Teng, 2019). This balance ensures that the app is educational and enjoyable and enhances user engagement and effectiveness. Lumosity (2024), a mHealth app designed to enhance memory, mental agility, and problem-solving skills, achieves this balance. The app uses personalized activities to match the user's abilities. It also includes built-in feedback and progress tracking to promote engagement and motivation further (Lumosity, 2018).

2.6 "Understand the emotion your game is trying to evoke"

Rosewater explained, "To be successful with your game, you need to know what your audience is trying to experience… everything in the game has to contribute to the emotional output you're trying to create" (GDC, 2016). This principle emphasizes the importance for clinicians and app designers to create features that elicit emotional responses, guiding users toward their desired mental health outcomes. If an app aims to elicit positive emotions, its features should be designed to maximize these outcomes. Feelings of surprise, humor, calm, and warmth can be fostered through text-based

narratives, sound prompts, and visual design. Features should be consistently evaluated and refined to ensure they contribute to the intended emotional experience.

For instance, an app that promotes positive emotions could include a daily gratitude journal. Each time a user records something they are grateful for, the app can provide positive reinforcement through uplifting quotes, cheerful animations, or encouraging sound effects. This feature taps into the emotion of gratitude and reinforces it with elements designed to make users feel appreciated and motivated.

Another example would be an app designed to foster calm and relaxation. The app might incorporate guided mindfulness and relaxation exercises. These sessions could use soothing background music, serene visual imagery, and gentle narration. By consistently evoking a sense of peace and relaxation, the app could help users manage stress and anxiety and move them closer to their mental health goals. Regular updates and user feedback would ensure these features remain effective and engaging (Luxton et al., 2011).

2.7 "Allow the player the ability to make the game personal"

In MTG, "land" cards function as a basic resource used to generate mana, the game's equivalent of action points needed to play other cards and abilities. In initial game versions, land cards had basic artwork and design with little variation. However, the game designers began experimenting with different versions of the land cards and adapted them to feature a variety of fantasy landscapes and realistic locations (Dunn, 2024). This allowed players to enhance their personal connection to the game and choose and play with their favored designs. Offering users choices, even those that do not alter the underlying mechanics, fosters personalization, which drives engagement (Ammenworth et al., 2023). Rosewater explains that familiarity breeds preference, and preference enhances perceived quality: "The more your players feel the game is about them, the better their brain will think of it" (GDC, 2016). Providing a user with numerous choices, resources, paths, expressions, and personalization options can maximize their engagement.

An app designed to improve mental well-being could offer personalized goal-setting features. Users could set mental health goals, such as practicing mindfulness, completing daily exercises, or tracking mood changes. The app could provide various tracking methods, like journals, visual progress charts, and motivational messages tailored to the user's preferences. This customization ensures that users feel the app addresses their unique needs and goals, fostering a deeper connection and commitment to their mental health journey.

2.8 "The details are where your player falls in love with the game (details matter)"

Mark Rosewater emphasizes the importance of details in game design: "As players explore your choices, they are searching for things to bond with. The players want to find a piece of the game to call their own" (GDC, 2016). Game details help explain many aspects of the game and allow players to explore connections. While not every detail will matter to every player, the smallest detail may be significant to some game users. Images and text that resonate with users' experiences help them explore connections and form meaningful bonds to the game or app. The bond enhances their drive to continue using the app and makes the experience more personal. Moreover, it encourages users to see and track the outcomes of their efforts and progress. Including detailed information and images can further increase continued app usage and deepen the user's engagement with the mHealth app to help them fall in love with it.

Applied to mHealth, an app can be designed to include personalized content and micro-interactions that reflect the user's preferences and behaviors. For example, if the app tracks a user's mood, it could offer tailored tips, inspirational quotes, or affirmations based on their current emotional state. These small, detailed interactions indicate that the app is attentive to the user's unique journey and fosters a sense of care and personalization that encourages continued use.

Another example would be to provide users with detailed feedback on their progress, which could help them form a deeper connection with the app. To illustrate, an app designed to support mental health could offer comprehensive summaries of the user's achievements, overcome obstacles, and identify patterns observed over time. Visual elements like graphs, charts, and personalized messages that celebrate milestones can make the user feel recognized and valued, reinforcing their commitment to the app and pursuit of their mental health goals.

2.9 "Allow players to have a sense of ownership (through customization)"

Rosewater's ninth lesson emphasizes the significance of customization. MTG fosters inclusivity by allowing the players to customize their decks in ways that reflect their personal identities. Each card represents different versions from which players can choose and make their decks a personal extension of themselves. When a player's customized deck wins, it feels like a personal victory (GDC, 2016).

Similarly, in mHealth, designers and clinicians should focus on creating inclusive and customizable app designs. An app should be user-friendly, easily accessible, and simple for users to modify. Personalization options can significantly enhance the treatment experience by allowing users to tailor the app to their unique needs and preferences (Lazard et al., 2021). For instance, users could choose from different meditation techniques, mental health exercises, or educational materials that resonate with their preferences. A mHealth app could also allow users to set and customize their mental health goals and treatment plans. Users might choose specific focus areas, such as stress reduction, improved sleep, or enhanced physical activity. The app could then provide tailored suggestions, resources, and reminders based on these preferences. By focusing on customization and providing a wide range of customizable content, mHealth apps can cater to diverse user needs, empower users, foster a sense of ownership, and enhance the overall effectiveness of the treatment provided.

2.10 "Leave room for your players to explore"

In Lesson #10, Rosewater highlights the importance of allowing players to feel more invested in experiences they initiate themselves. This strategy promotes user development by providing them with "room to grow" and emphasizes infrastructure that supports user adaptability and responsibility (GDC, 2016). In mHealth, this principle can enhance mental health outcomes by fostering a sense of exploration and self-directed growth between clients and healthcare providers.

As mHealth services and technology converge, creating flexible and adaptable mHealth platforms can significantly enhance client experiences (Ahern et al., 2011). Incorporating personalized features such as novel emotional regulation techniques, mental health daily maintenance tracking, and wearables can advance the harmonization of biofeedback systems with mental health management. An adaptable mHealth platform can serve to increase clients' investment in treatment decisions and foster a personalized approach to improving patient quality of life, symptom management, and mental health treatment adherence (Alessa et al., 2018; Whitehead & Seaton, 2018).

For instance, a mHealth app could offer a dynamic goal-setting feature where users can set, adjust, and track their mental health goals, similar to inpatient portals that allow for hospital customizations (McAlearney et al., 2019). This feature might include adaptive suggestions based on user progress and allow continuous growth and adaptation. For example, if a user sets a goal to meditate daily, the app could provide feedback and incorporate new challenges as the user develops their practice. This flexibility empowers users to take ownership of their mental health journey and contribute to a deeper commitment to their goals.

Similarly, integrating wearable technology that provides biofeedback, such as heart rate variability or sleep patterns, can enhance the adaptability of the mHealth app (Gordon et al., 2020). Users could receive real-time feedback and personalized recommendations based on their physiological data. This feature allows users to explore how their behaviors impact their mental health and make informed decisions about their treatment. A mHealth app like Calm (2024), allows users to connect with their mental and physical health in real-time by using the Apple Watch or other smart watch devices to track biometrics like heart rate variability and other personalized feedback. By offering a personalized and responsive platform, the app can increase user engagement and adherence to treatment.

2.11 "If everyone likes the game, but no one loves it, it will fail"

This lesson extends the concept of mHealth personalization described in Lesson #10. As technology advances and innovation increases, mHealth applications have shown substantial potential to be user-modifiable and adaptable. Unique methods of client engagement can personalize mHealth experiences and improve quality of life by building a framework tailored to each client's needs (Grossman et al., 2020). Configurable settings in mHealth applications allow clients to individualize their experience and make it reflective of their unique preferences and clinical needs. An evolving user platform provides clients with the ability to be actively engaged in treatment decision-making processes.

To illustrate, a mHealth app could create a more engaging experience by incorporating personalized community features where users can connect with others who share similar experiences and challenges. Features like discussion forums, group challenges, and peer support systems help build a sense of belonging and community. Community feature engagement may also facilitate user motivation, medication compliance, and treatment adherence by fostering supportive networks and optimal social comparisons (Cahn et al., 2018). This provides emotional support and increases user engagement and satisfaction, as they feel part of a supportive network that enhances their journey towards better mental health.

2.12 "Don't design to prove you can do something"

Rosewater comments, "It's not about enjoying the experience or testing yourself but rather about making the best game possible" (GDC, 2016). He emphasizes that designers should create with the user in mind rather than to prove their capabilities. Applications should reflect core principles prioritizing the user's experiences over the designer's desire for recognition or enjoyment. When designers and clinicians focus more on building something for their own recognition, the user's experience can suffer, and the app may lack essential components.

To optimize the user experience in mHealth apps, designers and clinicians must formulate app designs with the target audience in mind to ensure the inclusion of meaningful elements that enhance the user experience and effectively support behavioral health improvement.

Implementing iterative testing and feedback loops can help refine the app based on real user experiences (Huguet et al., 2023). Beta testing with a diverse group of users allows for the collection of feedback on usability, functionality, and overall satisfaction (Cho et al., 2024). Continuous updates and improvements based on this feedback ensure that the app evolves to meet user needs effectively and focuses on delivering the best possible experience rather than just showcasing advanced features.

2.13 "Make the fun part also the best strategy to win"

Traditional therapy uses interventions to explore new techniques that can enhance an individual's outlook, but these treatment approaches are considered to be still in its infancy and do not incorporate recent technological advancements (Rief, 2021; Goldfried, 2021). mHealth applications can supplement topics covered in therapy with psychoeducation and providing the user with support and engagement. In MTG, the mechanic of retrieving lost cards was designed to reignite the thrill of the game and allowed players to follow the game's guidance to lead to an enjoyable experience. This principle can be applied to mHealth apps: even challenging treatments should offer a sense of achievement and enjoyment. The fun aspects should be easily accessible and integrated into the core experience to ensure users find pleasure and success in their journey.

A mHealth app could incorporate reward-based activities where users earn points or badges for completing therapeutic tasks, such as meditation sessions or journaling. These rewards not only make the tasks more enjoyable but also motivate users to continue engaging with the app, reinforcing positive behavior through fun incentives.

2.14 "Embrace bluntness for audience understanding"

Rosewater likened bluntness to a "hammer in a creative toolbox" (Rosewater, 2016), which emphasizes its effectiveness in delivering a clear and directed message. In contrast, vague information can lead to various interpretations, potentially obscuring the intended message, and subtlety may result in critical information being overlooked. Therefore, when designing mHealth applications, developers and clinicians should prioritize clarity to ensure patients fully comprehend the information, objectives, or tasks presented.

Research indicates that succinct, comprehensible, and easily accessible information can enhance patients' understanding of their disease, symptoms, and medication (Vo et al., 2019). This clarity fosters confidence in patients' knowledge of their condition. Additionally, collaboration between app developers and health are providers is essential in creating applications that track individual growth and clearly outline the steps needed for improvement. This approach can significantly enhance patient engagement and treatment outcomes.

For example, if an app provides educational content, it should be concise and to the point. Instead of lengthy articles, the app might use bullet points, infographics, and short videos to convey essential information about mental health conditions and treatments. This approach ensures that users quickly grasp important concepts without feeling overwhelmed and can improve their knowledge and confidence in managing their health.

2.15 “Design the component for your intended audience”

After twenty years of developing MTG, Rosewater highlights the importance of identifying the player audience through psychographic data analysis (GDC, 2016). Psychographics, often used in marketing, examine factors driving consumer behavior (Barber et al., 2012). By utilizing a "psychographic profile," MTG designers classify the target audience into one of three "characters": Timmy, Johnny, and Spike (Rosewater, 2016). These characters represent different audience segments and provide developers with insights to align game features with consumer interests. Designers use methods such as focus groups, website analytics, and social media activity to understand player profiles.

Similarly, behavioral health professionals use a comprehensive approach to identify client populations. Case conceptualization is a framework for assessment and treatment planning in clinical practice (Ridley & Jeffrey, 2017). This process involves clinician insight and consideration of relevant theoretical, empirical, and cultural factors to analyze maladaptive behaviors and develop an ongoing comprehensive client profile to enhance treatment outcomes (Christon et al., 2014). Case conceptualizations have proven effective in treating non-suicidal self-injurious behavior (Andover et al., 2012), depression, and anxiety (Birdsey, 2020).

To maximize the useability of mHealth applications, behavioral health and gaming professionals should emphasize the idiographic measure of case conceptualization by 1) identifying user-specific risk and precipitating factors that may moderate maladaptive behaviors and daily functioning, and 2) highlighting possible protective factors, such as identifying the user's strengths and resiliency that may influence treatment outcomes.

A mHealth app could utilize psychographic profiling to tailor content delivery to individual users. Similarly, the app could incorporate adaptive learning algorithms that adjust the difficulty and type of therapeutic exercises based on the user’s progress and engagement levels. By continuously analyzing user behavior and feedback, the app can provide a personalized therapeutic experience that aligns with the user’s specific needs and preferences.

2.16 “Be more afraid of boring your players than challenging them”

mHealth applications have the potential to offer unique behavioral health interventions and strategies distinct from traditional treatment methods (Jeon et al., 2024). While novel approaches require rigorous testing to determine acceptability, feasibility, and efficacy, mHealth techniques can present established interventions in more dynamic and appealing ways. The high potential for innovation presents both challenges and opportunities for growth. Traditionally driven by creating gold-standard treatments with proven clinical effectiveness, the behavioral health field often progresses slowly. In contrast, the rapid release of new mHealth apps offers users a wide range of options, though it can also lead to difficulties in selecting the most beneficial apps (Wasil et al., 2021). Instead of viewing this as a barrier, the creative potential of diverse applications should be harnessed to provide users with novel treatment options that often may not be available with traditional behavioral health treatment.

An example of such novel treatments is interventions based on virtual reality (VR). A mHealth app could integrate VR to present controlled exposure scenarios for treating anxiety disorders. Additionally, VR techniques can provide an immersive and engaging experience that traditional methods may lack.

2.17 “You don’t have to change much to change everything”

In MTG, "five colors of magic" are represented on a color wheel (Sabiens, 2024). Initially, players were encouraged to use multiple colors while playing cards, but a later edition promoted using the fewest colors possible. This subtle change significantly impacted user engagement and demonstrated that minor adjustments can have profound effects. Similarly, small changes to an existing mHealth app can significantly increase user engagement and effectiveness. Adjustments such as customizing content to appeal to a different user base can enhance the app's impact without necessitating a complete redesign (Carlo et al., 2019).

For example, a mHealth app could offer settings to tailor content to different ethnicities and cultural backgrounds. Providing meditation guides in various languages or featuring culturally relevant examples could make the app more inclusive and engaging.

2.18 “Restrictions breed creativity”

Mark Rosewater’s use of the word “restriction” does not imply limiting what people can and cannot do; rather, it refers to narrowing the focus to a simpler subject or objective. This focused approach allows for a thorough evaluation and exploration of new perspectives, fostering creativity. In many ways, it encourages the development of innovative ideas that might not emerge from a broader focus. App developers and healthcare providers can leverage restrictions to enhance mHealth apps by concentrating on one feature at a time and finding novel ways to improve efficiency and user experience. Restrictions are a valuable tool for developing outstanding mHealth apps, guiding creators to establish unique and productive features compared to other similar mHealth apps.

For instance, a mHealth app could focus on tracking a single symptom, such as anxiety, rather than a broad range of mental health issues. By restricting the focus, developers can create detailed, tailored tracking tools and interventions that provide users with specific, actionable insights. Conversely, developers might concentrate on perfecting one key feature, such as a meditation guide. By dedicating resources to optimizing this feature, they can incorporate innovative techniques, such as biofeedback, to enhance its effectiveness and user engagement.

2.19 “Your audience is good at recognizing problems and bad at solving them”

Incorporating audience feedback is a powerful resource for mHealth developers. Users can provide insights into latent factors underlying successful mHealth interventions, although they may not always offer solutions. Establishing executive boards composed of content experts, stakeholders, and individuals with lived experience can provide valuable information on mHealth app development. These experts’ insights can also promote cross-sector collaborations to identify unique dynamics that contribute to successful mental health treatment outcomes. Future research should focus on creating networks of content experts to inform mHealth app development and isolate variables that hinder optimal mental health outcomes.

One possibility to accomplish such a goal is creating user advisory panels that provide regular feedback. This can help developers to stay attuned to user needs and preferences. These panels can highlight areas for improvement that the developers might not have considered, such as the need for more culturally diverse content.

2.20 “All lessons are interconnected”

All the lessons discussed in this paper are interconnected, each representing a part of a larger framework for creating an effective mHealth app. These lessons should be viewed not as isolated steps but as components of a cohesive strategy aimed at making the app as engaging and beneficial as possible. An aesthetically pleasing app will attract users and encourage continued use, leading to deeper exploration and interaction with the app’s features. The interconnected nature of these lessons means that collectively, they can help behavioral health providers offer comprehensive virtual resources that improve the users' lives globally.

3 AI-Assisted Inductive Thematic Analysis

Input Prompt #1

Perform an inductive thematic analysis on the attached transcript of Mark Rosewater's presentation titled "Magic: the Gathering: 20 Years, 20 Lessons Learned" and the attached integrative summary titled "Magic: The Gathering' and mHealth: Game Design Principles." The results should be provided in a with at least two specific examples drawn from the documents to illustrate the themes created. Additionally, provide a text summary of the results suitable for inclusion in an academic journal.

Output #1

An inductive thematic analysis of Mark Rosewater's "Magic: The Gathering: 20 Years, 20 Lessons Learned" transcript and the integrative summary "Magic: The Gathering' and mHealth: Game Design Principles" reveals three key themes: User-Centric Design, Emotional Engagement, and Structural Integrity in Design. This analysis highlights the interconnected principles that are crucial for creating engaging and effective experiences, whether in a tabletop card game or a mobile health application.

Thematic Analysis

Theme 1: User-Centric Design

This theme emphasizes that the user's perspective, preferences, and inherent behaviors are central to successful design. The design should adapt to the user, not the other way around. This involves deep empathy and a commitment to creating an experience that feels intuitive and personalized.

- Example 1: Aligning with Human Nature Mark Rosewater explains that "fighting against human nature is a losing battle". He recounts how game designers initially prevented a creature card from attacking immediately after being played, a rule that players instinctively ignored. The eventual solution was to change the rules to align with this "human nature". The mHealth summary applies this by suggesting that if smartphone users are accustomed to swiping to navigate, a health app should incorporate these familiar gestures rather than forcing users to learn new, non-standard controls.
- Example 2: Customization and Ownership Rosewater highlights how allowing players to customize their decks fosters a sense of personal ownership; when a personalized deck wins, "it feels like a personal victory" because the deck is an "extension of themselves". This is mirrored in the mHealth context by suggesting that apps should allow users to set and customize their own mental health goals and treatment plans. By choosing focus areas like stress reduction or improved sleep, users feel the app is tailored to their unique needs, fostering a deeper commitment.

Theme 2: Emotional Engagement

This theme focuses on the importance of creating a strong emotional connection with the user. A successful design goes beyond functionality to evoke feelings, resonate with personal experiences,

and create a memorable and enjoyable interaction. The goal is to make users "love" the experience, not just like it.

- Example 1: The Power of Resonance Rosewater uses the concept of "resonance," explaining that designers can tap into players' pre-existing emotional connections with concepts from pop culture, like zombies. The mHealth summary connects this to "empathic resonance" in psychotherapy and suggests that apps can achieve this by allowing users to create personalized avatars or storylines that reflect their aspirations. The app *Zombies, Run!* is cited as an example where storytelling is blended with fitness goals to make the experience more engaging.
- Example 2: Evoking Specific Emotions Rosewater stresses the need to "understand what emotion your game is trying to evoke" and ensure every element contributes to that feeling, whether it's the "terror" of a horror-themed set or the fun of a humorous one. The mHealth summary translates this by explaining that a relaxation app like Calm uses "soothing colors, balanced design elements, and gentle animations" to create a calming environment. Similarly, a gratitude journal feature could use uplifting quotes and cheerful animations to reinforce feelings of appreciation and motivation.

Theme 3: Structural Integrity in Design

This theme addresses the underlying framework and mechanics of the design. It posits that a well-structured game or application provides clarity, directs users toward beneficial behaviors, and allows for both focused creativity and emergent discovery. The design's "bones" must be sound to support a positive user experience.

- Example 1: Making the Fun Part the Best Strategy Rosewater warns against designs where the optimal way to win is not fun, citing a mechanic that discouraged players from talking or interacting to avoid penalties. The principle is that the "fun part" should also be "the correct strategy to win". In mHealth, this translates to integrating enjoyable elements into therapeutic tasks. For example, a reward system with points or badges for completing meditation sessions makes the tasks more enjoyable and motivates continued engagement, reinforcing positive behavior through fun incentives.
- Example 2: Restrictions Fostering Creativity Rosewater argues that "restrictions breed creativity" by forcing designers to solve problems in novel ways. He contrasts the difficulty of designing for an open-ended prompt with a themed one, where the constraints push him down new creative paths. The mHealth summary applies this by suggesting that developers can create superior features by narrowing their focus. For instance, an app could concentrate on perfecting a single feature, like a meditation guide with biofeedback, or restrict its focus to tracking a single symptom, allowing for more detailed and tailored tools.

Summary for an Academic Journal

An inductive thematic analysis was performed on the transcript of Mark Rosewater's 2016 Game Developers Conference presentation, "Magic: The Gathering: 20 Years, 20 Lessons Learned," and an

integrative document applying these lessons to mobile health (mHealth) design. The analysis revealed three salient, interconnected themes: User-Centric Design, Emotional Engagement, and Structural Integrity in Design.

The theme of User-Centric Design underscores the principle that effective design must be congruent with users' innate behaviors, cognitive patterns, and desire for personalization. Both sources emphasize adapting the design to the user, rather than compelling the user to adapt to the design, through features like intuitive controls and extensive customization options that foster a sense of ownership.

The second theme, Emotional Engagement, highlights the criticality of evoking a strong, positive emotional response. Successful design transcends mere utility by creating resonance with users' experiences, memories, and values. This is achieved through aesthetic considerations, storytelling, and a clear focus on the specific emotions the experience is intended to elicit, with the ultimate goal of creating a product that users "love" rather than merely "like."

Finally, Structural Integrity in Design pertains to the underlying mechanics and framework that guide the user experience. This theme suggests that the most effective path to achieving goals should also be the most enjoyable. It also posits, counterintuitively, that constraints and limitations are not obstacles but catalysts for creativity, leading to more innovative and focused design solutions.

Collectively, these themes suggest a holistic design philosophy applicable across disparate fields like gaming and mHealth, where success is predicated on a deep understanding of human psychology, a commitment to emotional connection, and a robust and intentional design structure.

Input Prompt #2

Repeat the inductive thematic analysis on the attached transcript of Mark Rosewater's presentation titled "Magic: the Gathering: 20 Years, 20 Lessons Learned" and the attached integrative summary titled "Magic: The Gathering' and mHealth: Game Design Principles." Use the following two themes: 1) gamification defined as integrating game elements and principles into mobile health applications to enhance user engagement and motivation, and 2) socialization defined as promoting social experience and connection. The results should be provided with at least two specific examples drawn from the documents to illustrate the themes. Additionally, provide a text summary of the results suitable for inclusion in an academic journal.

Output #2

An inductive thematic analysis of Mark Rosewater's "Magic: the Gathering: 20 Years, 20 Lessons Learned" transcript and the "Magic: The Gathering' and mHealth: Game Design Principles" summary, using the predefined themes of Gamification and Socialization, reveals how core principles from game design can be strategically applied to enhance user experience in non-game contexts like mobile health (mHealth).

Thematic Analysis

Theme 1: Gamification

Gamification is defined as integrating game elements and principles into mobile health applications to enhance user engagement and motivation. This theme explores how mechanics like rules, feedback, and reward systems can make challenging or mundane tasks more enjoyable and compelling, driving continued participation.

- Example 1: Making Fun the Correct Strategy Rosewater describes creating a humorous game set where the optimal strategy became decidedly unfun: to win, players had to avoid talking or laughing to prevent their opponents from gaining an advantage. The lesson learned was to "make the fun part also the correct strategy to win". The mHealth summary applies this by suggesting that even challenging therapeutic treatments should incorporate a sense of achievement and enjoyment. An mHealth app could implement this through reward-based activities, such as earning points or badges for completing tasks like meditation or journaling. This gamification principle ensures that engaging with the app's core therapeutic purpose is also a rewarding and motivating experience.
- Example 2: Leveraging Resonance and Fantasy Rosewater emphasizes the importance of resonance, where a game connects with a player's pre-existing knowledge and emotions, such as using familiar fantasy tropes like zombies. The mHealth summary highlights *Zombies, Run!*, an app that gamifies exercise by blending storytelling with fitness goals. Users follow a narrative, complete missions, and overcome obstacles as they run or walk, making the physical activity more engaging. This application of gamification creates emotional resonance by transforming fitness into an immersive experience, thereby boosting user motivation.

Theme 2: Socialization

Socialization is defined as promoting social experience and connection. This theme examines how design choices can foster a sense of community, facilitate interaction, and allow users to share their experiences, which can be a powerful motivator and support mechanism.

- Example 1: Player-Created Formats and Community Rosewater discusses the creation of Commander, a popular game format that was not designed by the company but by a group of players (judges) who wanted a unique way to play together after their work was done. This player-driven format was so popular that the company eventually began creating products to support it. This illustrates how allowing space for socialization and community-driven innovation can lead to deep and lasting engagement.
- Example 2: Fostering Community in mHealth The mHealth summary suggests that apps can create more engaging experiences by incorporating personalized community features where users connect with others who share similar challenges. Elements like discussion forums, group challenges, and peer support systems can build a sense of belonging and community. This socialization can facilitate motivation and treatment adherence by creating supportive networks for users, which provides emotional support and increases satisfaction with the app.

Summary for an Academic Journal

A targeted thematic analysis of Mark Rosewater's presentation on game design and its application to mobile health (mHealth) applications was conducted to examine the principles of Gamification and Socialization. Gamification, defined as the integration of game elements into non-game contexts to enhance motivation, and Socialization, defined as the promotion of social connection, were found to be central pillars for driving user engagement.

The analysis of Gamification reveals that successful engagement hinges on structuring activities so that the most effective path to a goal is also the most enjoyable. Drawing from examples in game design where optimal but unfun strategies led to poor player experiences, the application in mHealth involves embedding reward systems, such as points or badges, directly into therapeutic tasks. Furthermore, leveraging narrative and fantasy, as seen in fitness apps that use storytelling to motivate exercise, demonstrates that gamification is most effective when it creates emotional resonance and transforms routine activities into compelling experiences.

The theme of Socialization highlights the importance of community and shared experience in sustaining user engagement. The organic development of player-created formats in gaming, which were later officially supported due to their popularity, underscores the power of user-driven social innovation. In the mHealth context, this principle translates to the deliberate inclusion of features like peer support forums and group challenges. These elements foster a sense of community and belonging, which can serve as a crucial support mechanism, thereby enhancing motivation and adherence to health-related goals. The analysis concludes that the thoughtful application of these two themes is critical for creating effective and engaging mHealth interventions.
